# Theatrical Performances at the New York State Lunatic Asylum

**Published:** 1847-04

**Authors:** D. Tilden Brown

**Affiliations:** 2d Assistant Physician


*Theatrical Performances at the New York State Lunatic Asylum.* By D. Tilden Brown, M. D., 2d Assistant Physician.—Among the moral agents employed in all well-regulated Asylums for the insane, to divert the deranged mind from its morbid concentration, by giving new direction to thought, and suggesting new sources of contemplation, amusements deservedly hold a high rank; and every appliance which conduces to gratify or create a capacity for enjoyment in the unhappy lunatic, however humble in itself, becomes a valuable adjuvant in the process of restoration. Varied as it may be by periodical diversions and occasional festivals, the hospital residence of the insane is necessarily, in a degree, monotonous and insipid. Separated by affliction from the world, with its ties, affections, and pursuits; excluded by misfortune from the usual sources of rational happiness, much of their life is passed in the gloom of despondency; in inward struggles with their emotions; in revolving cherished delusions; or in groping amid the darkness of fatuity. In removing or mitigating such conditions, mirth and recreation, no longer frivolousor puerile, become dignified as instruments of cure. They not only prove serviceable by presenting temporary enjoyment, but by creating a future in the mind absorbed in gloomy retrospect, or present wretchedness, and by furnishing cheerful attractions to the memory of those who drown all recollections of the past in apprehensions of impending evils. They impel the patient to the exercise of self-control, both by suggesting that propriety of behaviour which will secure participation in such privileges, and by engaging the attention to the exclusion of irrational thoughts. Though by some miserable victims of hopeless despair, all attempts to arouse their cheerfulness are viewed as cruel mockery of their sufferings, to many, such occasions are sources of unalloyed pleasure; and the aggregate gratification experienced by the insane in any entertainment, is probably but little less than would be manifested by an equal number of their more sane brethren.

With such an estimate of their value, the guardian of the insane seeks to accumulate sources of amusement that he may alleviate confinement and ennui, and at the same time so vary recreation that it may not satiate by its monotony. He thus opens a new world of pleasure to his charge, presenting enjoyments adapted to their mental condition, and their capacity to appreciate them. The character of these amusements is varied by manifold causes. Thus the whole system of recreation of any Asylum, may be influenced or decided by the temperament of its Superintendent; by the social position and former occupation of its inmates; or by the locale of the institution itself; while numerous minor agencies will affect the details of its operation. As a class, the amusements of the American Asylums rarely differ in a material degree, but a few find both advocates and opponents among the medical officers. Dancing, social parties, permitting the commingling of the sexes, fairs, and attendance on public entertainments, have been successively opposed and defended. When, however, it is remembered that many of the insane are but little separated from the rest of the world in their sense of propriety and correctness of deportment, there seems no reasonable objection, provided the indulgence be limited to this class, to a participation in those amusements which employ so much of the time of the sane world.

In this country, where the utilitarian spirit of the national character exhibits itself, even under the disturbing influences of mental alienation, employment in industrial pursuits, as a moral agent in the treatment of this disease, proves far more efficacious, and in general, more alleviating to the patient than mere recreation. In northern latitudes, however, such occupation is generally confined, by the severity of climate, to a comparatively small portion of each year; and although some branches of industry employ a limited number of patients during the tempestuous season, reliance is mainly placed on those ornamental arts and intellectual diversions which may be comprised in the term amusements. But these, of themselves, become agreeable and inviting occupations, arousing the inert, and tranquillizing the irritable. In their nature, they should be such as will engage, without exciting, those to whom they are addressed; and, while permitting that restraining surveillance which their conditions require, will insure cheerfulness and mirth. Of the numerous source of amusement, combining present gratification and prospective benefit, which are available in an Asylum, none, perhaps, presents more decided advantages than dramatic representation. All the necessary *materiel* exists within the precincts of the institution itself, requiring only to be developed and set in operation. Among the throng which fill its halls, move the author and the actor, waiting only to be discovered by the discriminating tact of their guardians. There is the genius to create, and the talent to pourtray scenes of humor, sentiment, and pathos. From such convictions, the attempt has been made at the State Lunatic Asylum, during the present winter, to produce the exhibition of theatrical performances by the patients, for the twofold purpose of engaging some who pertinaciously object to all other employment, and offering an agreeable diversion to many others. These efforts have been attended with gratifying success, and the representations have realized the most sanguine hopes of the projectors. Original plays have been produced and enacted solely by the patients, and the approval of competent judges has pronounced the matter and the impersonation to be meritorious. The hope of approbation undoubtedly stimulated some to engage in this project, who might otherwise have declined; and the presence of the Managers of the Asylum with their families and friends, and, on one occasion, that of the Governor of the State, has gratified the desire, and fairly tested the feasibility of this enterprise. Although the performers consisted entirely, and the spectators mainly of patients, it is believed that an eyewitness of the scene, unconscious of these facts, would have discovered no indication thereof throughout the performance, or would have credited the assurance that the actors before him, and the spectators about him, were isolated from the world as victims of insanity.

The plays enacted thus far have been mostly humerous; caricatures of the follies of the age, burlesques on national characteristics, and ludicrous exhibitions of individual and sectional peculiarities. There can be little doubt, however, that with the assistance of suitable appliances, pieces of higher pretensions, vaudevilles, melodramas, &c., might be successfully attempted.

Some patients have evinced a felicity in the conception and impersonation of the characters assigned them, not unworthy of professed representatives of the histrionic art.

Seventeen patients have assisted in these performances, some of whom have since recovered and left the institution. Others are convalescing, while to a smaller number, the Asylum will probably always prove a better home than the world with its turmoil can offer. Among these were individuals who believed, or had recently believed themselves to be the especial ministers of Divine will; exalted personages, Roman Pontiffs, or Secretaries of State; that by a wave of their hand they could control the movements of railroad trains, and vessels on the high seas; who had, previous to admission, been confined in chains, as dangerous, from their homicidal propensities; who insisted on their own idiocy and "inability to think," even while engrossed in the study of their parts; or whose ordinary conversation denied all attempts to trace coherence or point.

No injurious effects have, thus far, been discovered, and none are apprehended from the continuance of these diversions, if proper discrimination in the selection of performers, and admission of spectators, be exercised. On the other hand, undoubted benefits have accrued from the intellectual application, mental discipline, exercise of memory, and self-control of the performers, and from the diffusion of good humor and hilarity among the observers. The success of this attempt to remove the barrier which has hitherto debarred the insane from enjoyments of this nature, thus assimilating their condition more nearly to that of their sane brethren, is alike gratifying to the philanthropist, interesting to the philosopher, and encouraging to those whose duty and pleasure it is to "minister to minds diseased."—*N. Y. Annalist.*

